# Efficacy and outcomes of antiplatelet therapy versus oral anticoagulants in patients undergoing transcatheter aortic valve replacement: a systematic review and meta-analysis

**DOI:** 10.1097/MS9.0000000000001908

**Published:** 2024-03-15

**Authors:** Aman Goyal, Fatima Qayyum Abbasi, Muhammad Daoud Tariq, Sai Gautham Kanagala, Mah I. Kan Changez, Darsh Safi, Jawad Basit, Samia Aziz Sulaiman, Mohammed Dheyaa Marsool Marsool, Mohamed Daoud, Amir H. Sohail

**Affiliations:** a Department of Internal Medicine, Seth GS Medical College and KEM Hospital, Mumbai, India; b Federal Medical College; c Foundation University Medical College, Islamabad; d Department of Surgery, Quetta Institute of Medical Sciences, Quetta; e Department of Internal Medicine, Rawalpindi Medical University, Rawalpindi; f Department of Internal Medicine, Metropolitan Hospital Center, New York, NY; g Department of Internal Medicine, School of Medicine, University of Jordan, Amman, Jordan; h Department of Internal Medicine, Al-Kindy College of Medicine, University of Baghdad, Baghdad, Iraq; i Department of Internal Medicine, Bogomolets National Medical University, Kyiv, Ukraine; j Department of Surgery, University of New Mexico Health Sciences, Albuqurque, NM

**Keywords:** antiplatelet therapy, cardiology, direct oral anticoagulants, TAVR, thrombotic complications, vitamin k antagonists

## Abstract

**Background:**

Recent guidelines suggest that antiplatelet therapy (APT) is the standard of care in the absence of long-term oral anticoagulation (OAC) indications in patients post-transcatheter aortic valve replacement (TAVR). The superiority of one method over the other remains controversial.

**Materials and methods:**

Several databases, including MEDLINE, Google Scholar, and EMBASE, were electronically searched. The primary endpoint was the all-cause mortality (ACM) rate. Secondary endpoints included cardiovascular death, myocardial infarction (MI), stroke/TIA, haemorrhagic stroke, bleeding events, systemic embolism, and valve thrombosis in post-TAVR patients receiving APT and oral anticoagulants (OACs). Forest plots were generated using Review Manager version 5.4, with a *p* value less than 0.05 indicating statistical significance. Subgroup analysis was performed to explore potential sources of heterogeneity.

**Results:**

Twelve studies were selected. No significant differences were observed in APT and OAC group for ACM [risk ratio (RR): 0.67; 95% CI:0.45–1.01; *P*=0.05], cardiovascular death [RR:0.91; 95% CI:0.73–1.14; *P*=0.42], MI [RR:1.69; 95% CI:0.43–6.72; *P*=0.46], Stroke/TIA [RR:0.79; 95% CI:0.58–1.06; *P*=0.12], ischaemic stroke [RR:0.83; 95% CI:0.50–1.37; *P*=0.47], haemorrhagic stroke [RR:1.08; 95% CI: 0.23–5.15; *P*=0.92], major bleeding [RR:0.79; 95% CI:0.51–1.21; *P*=0.28], minor bleeding [RR:1.09; 95% CI: 0.80–1.47; *P*=0.58], life-threatening bleeding [RR:0.85; 95% CI:0.55–1.30; *P*=0.45], any bleeding [RR:0.98; 95% CI:0.83–1.15; *P*=0.78], and systemic embolism [RR:0.87; 95% CI:0.44–1.70; *P*=0.68]. The risk of valve thrombosis was higher in patients receiving APT than in those receiving OAC [RR:2.61; 95% CI:1.56–4.36; *P* =0.0002].

**Conclusions:**

Although the risk of valve thrombosis increased in patients receiving APT, the risk of other endpoints was comparable between the two groups.

## Introduction

Highlights

- The risk of valve thrombosis was higher in patients receiving antiplatelet therapy as compared to oral anticoagulant (OAC) in patients post-transcatheter aortic valve replacement (TAVR).
- The risk of several other endpoints was comparable between the two groups.
- Post-TAVR treatment should be determined by considering factors such as the presence of indications for OAC use, risk of valvular thrombosis in the patient population, and presence of any contraindications to either drug class.

Transcatheter aortic valve replacement (TAVR) is a proven treatment for symptomatic aortic stenosis (AS)^1^. Recent trends favour TAVR over surgical valve replacement in the United States (72 991 versus 57 626 per year)^2^. There is a link between the increase in trend and the reduction in procedural complications over time, which in part can be attributed to the choice of the patient and the features of the device. TAVR indications are expanding, making it imperative to provide optimal medical care to post-TAVR patients to ensure favourable outcomes. It is noteworthy to mention the potential side effects following TAVR; 50% of strokes occur after the first day, and the risk of cerebrovascular events post-procedure extends up to 60 days. Although recent studies on TAVR have reported fewer bleeding events than traditional surgery, it is important to recognize these major bleeding events, as they are associated with adverse outcomes^3,4^. Recent consensus guidelines from experts recommend dual antiplatelet therapy (DAPT) for a duration of 3–6 months following TAVR^5^. For patients who do not have an indication for long-term oral anticoagulation (OAC), it is typically advised to start with DAPT, with low-dose acetylsalicylic acid at 75–100 mg once daily and clopidogrel at 75 mg once daily for a duration of 3–6 months. After this initial period, lifelong single-antiplatelet therapy (SAPT) is generally recommended. Current guidelines recommend the continuous use of vitamin K antagonists (VKAs) in patients with established atrial fibrillation (AF) undergoing TAVR. The challenges posed by VKAs, such as strict patient adherence, a narrow range of effective dosing, and interactions with different foods and medications, make their use challenging, especially for elderly patients with multiple health issues^6^. Current European and American guidelines recommend the use of direct oral anticoagulants (DOACs) as an alternative and are frequently employed in everyday clinical practice for TAVR patients requiring oral anticoagulation, although the data reported by studies represent inconsistencies between efficacies^7^.

Although several meta-analyses have compared the efficacy of DOACs and VKAs in patients post-TAVR, our comprehensive meta-analysis aimed to compare the efficacy and outcomes in patients after TAVR who received APT or OAC (either DOAC or VKAs) owing to the lack of comprehensive data and controversial opinions in the existing literature.

## Material and methods

This literature review and meta-analysis was performed in accordance with the guidelines provided by the Cochrane Collaboration^8^ and reported using the Preferred Reporting Items for Systematic Review and Meta-Analysis Statement (PRISMA 2020, Supplemental Digital Content 1, http://links.lww.com/MS9/A398)^9^. The protocol of the study was registered in the PROSPERO International Prospective Register of Systematic Reviews (CRD42023470549). The work has been reported in line with AMSTAR, Supplemental Digital Content 2, http://links.lww.com/MS9/A399 (Assessing the methodological quality of systematic reviews) Guidelines.

### Data sources and search strategy

An electronic search of the Cochrane Library, EMBASE, the international database of clinical trials (www.clinicaltrail.gov), MEDLINE (via PubMed), Google scholar, and Scopus from 15 September until 10 October, 2023. This search yielded a variety of study designs, from randomized controlled trials (RCTs) to observational studies. This study aimed to compare APT with OACs in patients who underwent TAVR. There were no time or language constraints in the search. Additionally, searches were conducted in the European Heart Journal, Circulation Research, and the Journal of the American College of Cardiology. To identify additional relevant studies, we also conducted a manual search of the reference lists. However, this did not result in the discovery of any suitable studies to be included. A manual search of reference lists was conducted to find more pertinent research. Predefined Medical Subject Headings (MeSH) terms in conjunction with the Boolean operators ‘AND’ and ‘OR’ were employed. The search strategy incorporated the following terms: ‘TAVI,’ ‘TAVR,’ ‘Transcatheter aortic valve implantation,’ ‘transcatheter aortic valve replacement,‘ ‘DOAC, ’NOAC,’ ‘direct oral anticoagulants,’ ‘anti-platelet therapy,’ ‘vitamin K antagonists,’ ‘warfarin,’ ‘APT. The detailed search strategy is mentioned in the Online Supplementary file, Supplemental Digital Content 3, http://links.lww.com/MS9/A400.

### Eligibility assessment

Two investigators independently reviewed the titles and abstracts for eligibility, and excluded studies that had duplicates or did not meet the inclusion criteria. After the initial screening, the full text of the included articles was studied to further exclude studies that did not adhere to predetermined eligibility criteria. The included articles were thoroughly studied to extract baseline characteristics, such as author, year, study design, follow-up period, number of patients, comorbidities, and other relevant characteristics. Disagreements were approached through a discussion initiated by a third reviewer to reach a consensus. No limitations were imposed on the sample size or follow-up duration.

### Eligibility criteria

The inclusion criteria were assessed using the Population, Intervention, Control, and Outcomes (PICOs) format for systematic reviews and meta-analyses. In our study, ‘P’ represented patients who had undergone TAVR therapy, ‘I’ represented patients taking antiplatelet therapy (APT), ‘C’ represented patients taking oral anticoagulants (OAC), and multiple outcomes were assessed, as discussed below.

Exclusion criteria^1^: studies that fit into any of the following categories: case reports, recommendations, literature reviews, systematic reviews, meta-analyses, letters to editors, or commentaries were disqualified; further exclusion criteria included^2^: patients with a history of bleeding disorders^3^; studies involving mechanical heart valve prostheses^4^; studies with any clear indication for DAPT^5^; studies using animal models; and^6^ studies lacking adequate clinical data pertinent to the outcomes being studied.

### Data extraction and quality assessment

Two researchers independently extracted the data from the studies included. In the event of disagreement, data were reviewed by an independent researcher. First author’s name, publication year, country, study design, sample size (active/control), intervention (dose/target), and reported outcomes. Quality assessment was performed using the modified Cochrane Collaboration risk of bias 2.0 tool for RCTs^8^ and the Newcastle–Ottawa scale for observational studies^10^.

### Endpoint definition

The primary endpoint was the all-cause mortality (ACM) rate. The secondary endpoints included cardiovascular death, myocardial infarction (MI), stroke/TIA, ischaemic stroke, haemorrhagic stroke, any bleeding, life-threatening bleeding, minor bleeding, systemic embolism, and valve thrombosis. These events were defined according to the criteria established by the Vascular Academic Research Consortium-2^11^ and Bleeding Academic Research Consortium^12^.

### Data synthesis

The extracted data were combined for meta-analysis using Review Manager (RevMan) Version 5.4 and above by two authors. Forest plots were plotted to evaluate dichotomous data with 95% CIs, and a *p* value of less than 0.05 was considered statistically significant. Random-effects meta-analyses were conducted for all outcomes, considering that different studies estimated varying intervention effects, which partially elucidates the heterogeneity between studies. For dichotomous data, the effect measure used was risk ratio (RR). Higgins I^2^ statistics were used to evaluate statistical heterogeneity, with a value of less than 25% considered low, 25–75% considered moderate, and greater than 75% considered high heterogeneity. Potential sources of heterogeneity were explored through subgroup analysis. The two subgroups were formed by differentiating studies based on their study design, with one subgroup including all RCTs and the other including all observational studies. Influential studies were assessed using sensitivity analysis.

## Results

### Study selection

**Figure 1:**
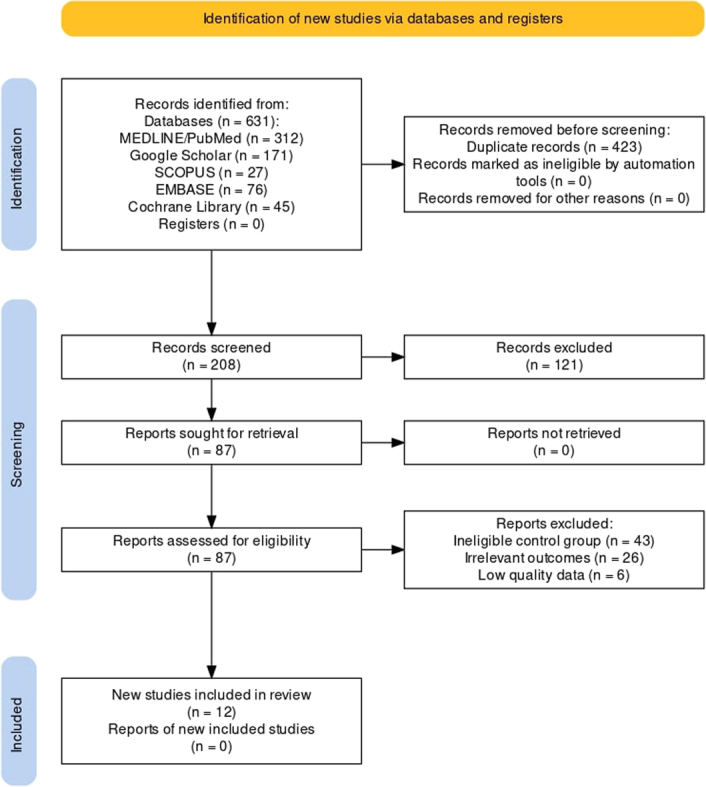
Preferred Reporting Items for Systematic reviews and Meta-Analysis (PRISMA) flow diagram for identification of studies included in the meta-analysis.

A total of 631 potentially relevant articles were identified after an extensive search across multiple databases. Among these articles, 423 were excluded based on preliminary screening. Articles excluded in the preliminary screening were due to data duplication, title and abstract not meeting our inclusion criteria. The full texts of the 87 articles were screened, of which were rejected due to (a) an ineligible control group (*n*=43), (b) irrelevant outcome measures (*n*=26), and (c) low-quality data (*n*=6). Finally, 12 studies^13–24^ were included in this meta-analysis. The detailed steps of the literature search are presented in the PRISMA flowchart (Fig. 1).

### Study and patient characteristics

Of the 12 studies included in the final analysis, 4 were RCTs and 8 were observational in design. A total of 7418 participants were included in this meta-analysis. Further details are presented in Table 1.

**Table 1:** Baseline characteristics and study details of all included studies.

			Age (mean years ±SD)		Male (*n*)		Hypertension (*n*)		Diabetes mellitus (*n*)		Congestive heart failure (*n*)		Cerebrovascular disease (*n*)		Coronary artery disease (*n*)		CHA2-DS2-Vasc Score	
References	Study design (*n*)	Sample size (*n*) (APT/OAC)	APT	OAC	APT	OAC	APT	OAC	APT	OAC	APT	OAC	APT	OAC	APT	OAC	APT	OAC
Collect *et al*.,^13^	RCT	751/749	82.3±6.4	81.6±6.1	360	344	601	606	214	221	284	292	89	78	NR	NR	4.3 ±1.4	4.4±1.4
Dangas *et al*.,^14^	RCT	818/826	80.8±6.0	80.4±7.1	405	426	697	720	235	236	380	394	35	51	305	325	NR	NR
Park *et al*.,^15^	RCT	118/111	80±5.3	80.2±5.2	47	49	84	81	36	35	12	17	11	6	34	32	NR	NR
Rogers *et al*.,^16^	RCT	50/44	73.1±5.7	73.6±4.0	37	29	39	36	15	17	NR	NR	1	1	NR	NR	NR	NR
De Backer *et al*.,^17^	Observational	116/115	80.5±6.2	79.7±7.3	74	74	95	98	27	21	95	98	6	11	36	42	NR	NR
Lee *et al*.,^23^	Observational	118/111	NR	NR	47	49	NR	NR	NR	NR	NR	NR	NR	NR	NR	NR	NR	NR
Kosmidou *et al*.,^18^	Observational	688/933	82.9±7.4	82.8±6.7	462	612	636	856	251	329	603	836	NR	NR	140	188	5.6±1.2	5.6±1.3
Gurevich *et al*.,^19^	Observational	90/101	82±10.6	81±11.3	65	93	NR	NR	NR	NR	32	24	8	21	NR	NR	NR	NR
Holy *et al*.,^20^	Observational	315/199	80.4 ± 7.0	80.6 ± 5.7	134	92	279	172	81	59	NR	NR	NR	NR	215	126	4.8±1.3	4.6±1.3
Nei *et al*.,^21^	Observational	193/571	83±7.6	82±7.4	110	318	172	521	79	217	NR	NR	36	89	461	383	4.5±1.3	4.8±1.3
Hohmann *et al*.,^22^	Observational	854/619	80.3±6.5	81.9±5.4	443	335	746	570	377	285	356	375	NR	NR	NR	NR	NR	NR
Poliacikova *et al*.,^24^	Observational	58/22	81.6 ± 6.4	NR	27	NR	NR	NR	NR	NR	NR	NR	NR	NR	NR	NR	NR	NR

APT, antiplatelet therapy; NR, not reported; OAC, oral anticoagulant; RCT, randomized controlled trials.

### Primary outcome

The ACM outcome was reported in eight studies^13–15,19,20,22^. No statistically significant difference was found between the APT and OAC groups with respect to ACM [8.35% versus 10.4%; RR:0.67; 95% CI:0.45–1.01; *P*=0.05; I^2^=76%] (Fig. 2A). To address heterogeneity, subgroup analysis was performed, and the heterogeneity reduced to nil in the subgroup containing only RCTs [RR: 0.60; 95% CI: 0.43–0.82; *P*=0.001; I^2^=0%] (Fig. 2B).

Figure 2(A–K) Individual pooled analysis illustrating the clinical efficacy of APT compared to vitamin K antagonist therapy. The risk ratio (RR) with their 95% CI are displayed using a logarithmic scale, with the box size scaling in accordance with the sample size. The diamond symbolizes the combined or overall effect. (A) All-cause mortality; (B) all-cause mortality (subgroup analysis); (C) death from cardiovascular; (D) death from cardiovascular causes (subgroup analysis); (E) myocardial infarction; (F) stroke/TIA; (G) stroke/TIA (subgroup analysis); (H) ischaemic stroke; (I) haemorrhagic stroke; (J) systemic embolism; (K) systemic embolism (subgroup analysis); (L) valve thrombosis; (M) valve thrombosis (subgroup analysis); (N) major bleeding; (O) major bleeding (subgroup analysis); (P) minor bleeding; (Q) Life-threatening bleeding; (R) life-threatening bleeding (subgroup analysis); (S) any bleeding. APT, antiplatelet therapy; OAC, oral anticoagulant.

**Figure:**
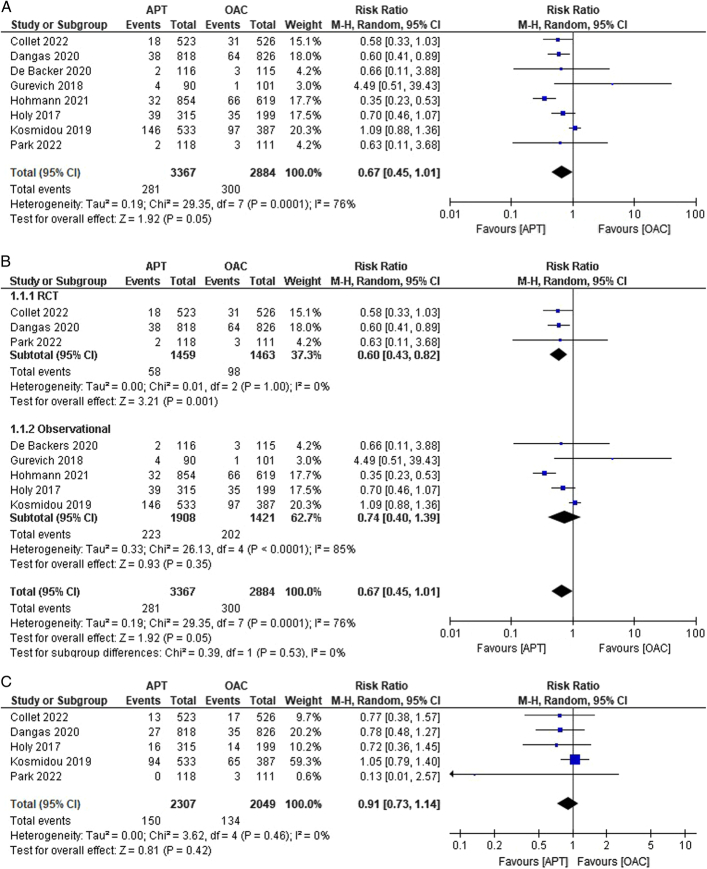

**Figure:**
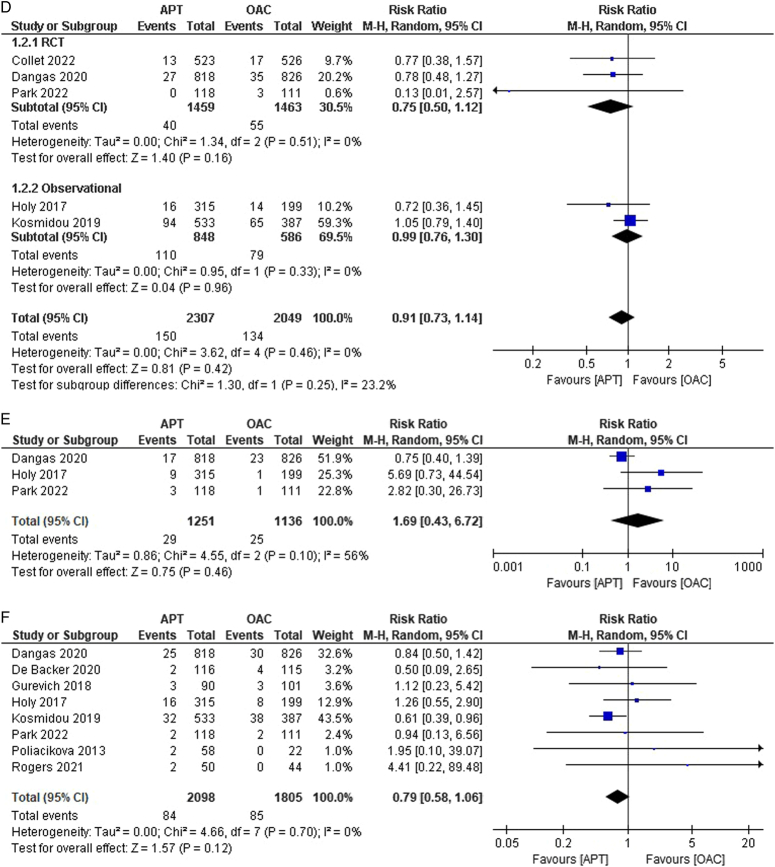

**Figure:**
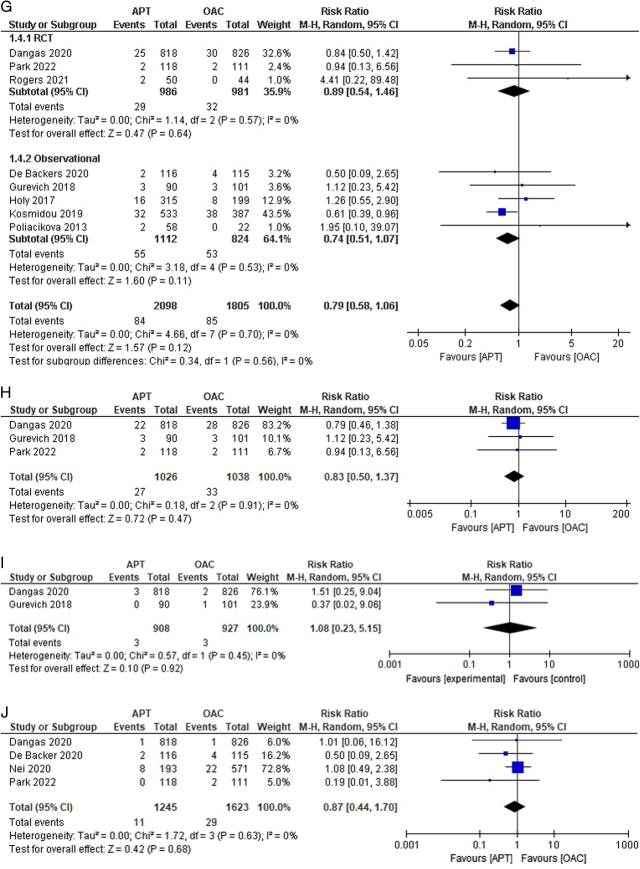

**Figure:**
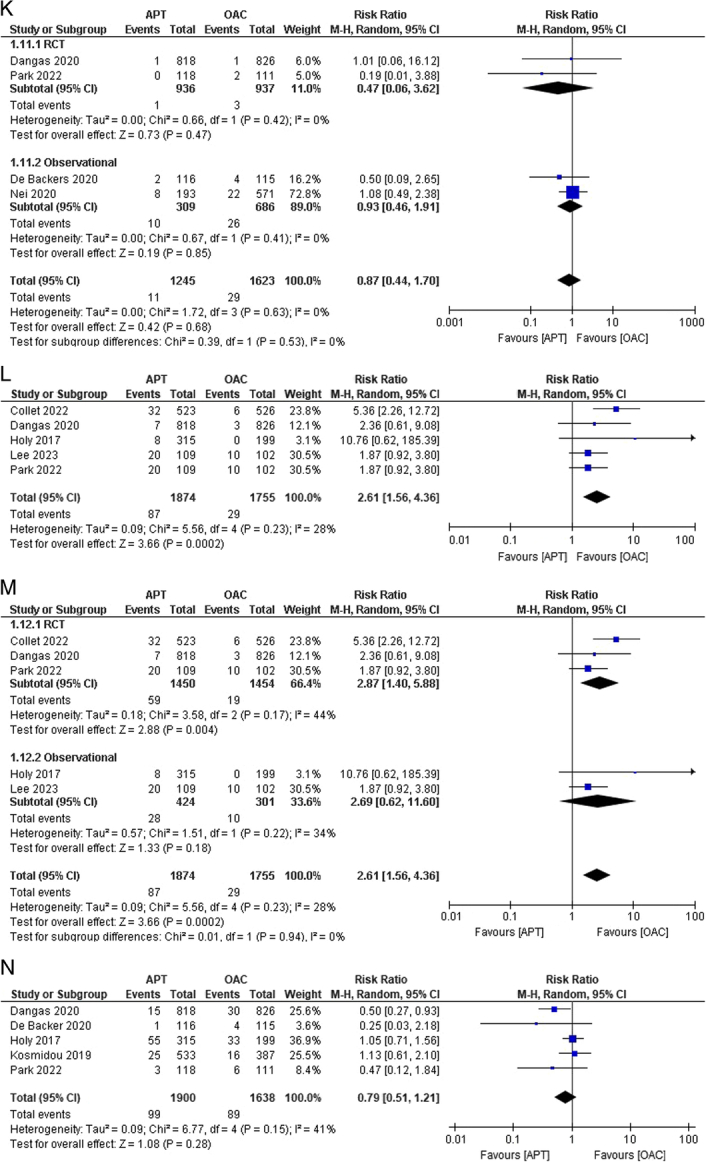

**Figure:**
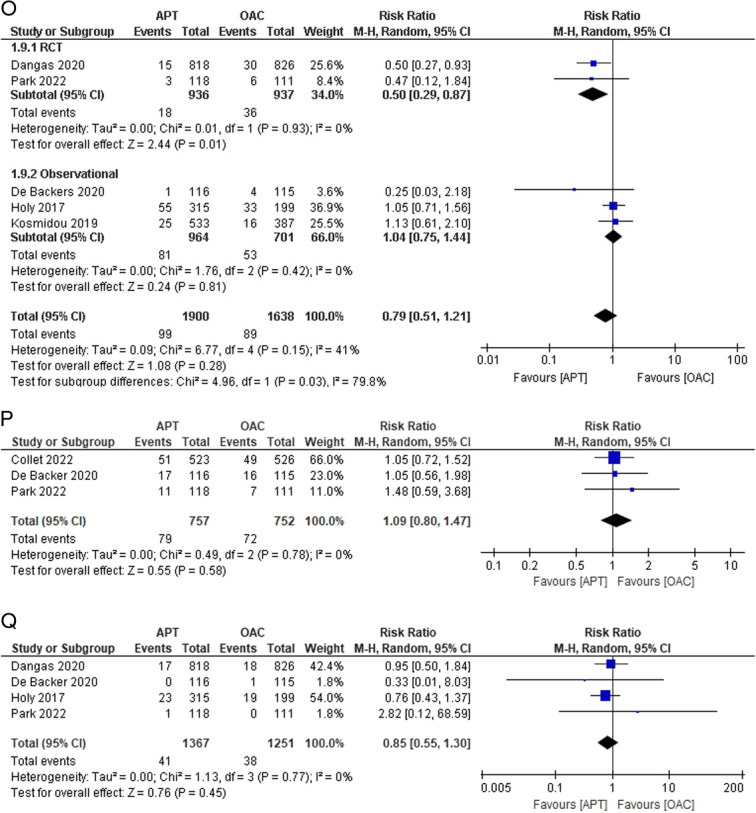

**Figure:**
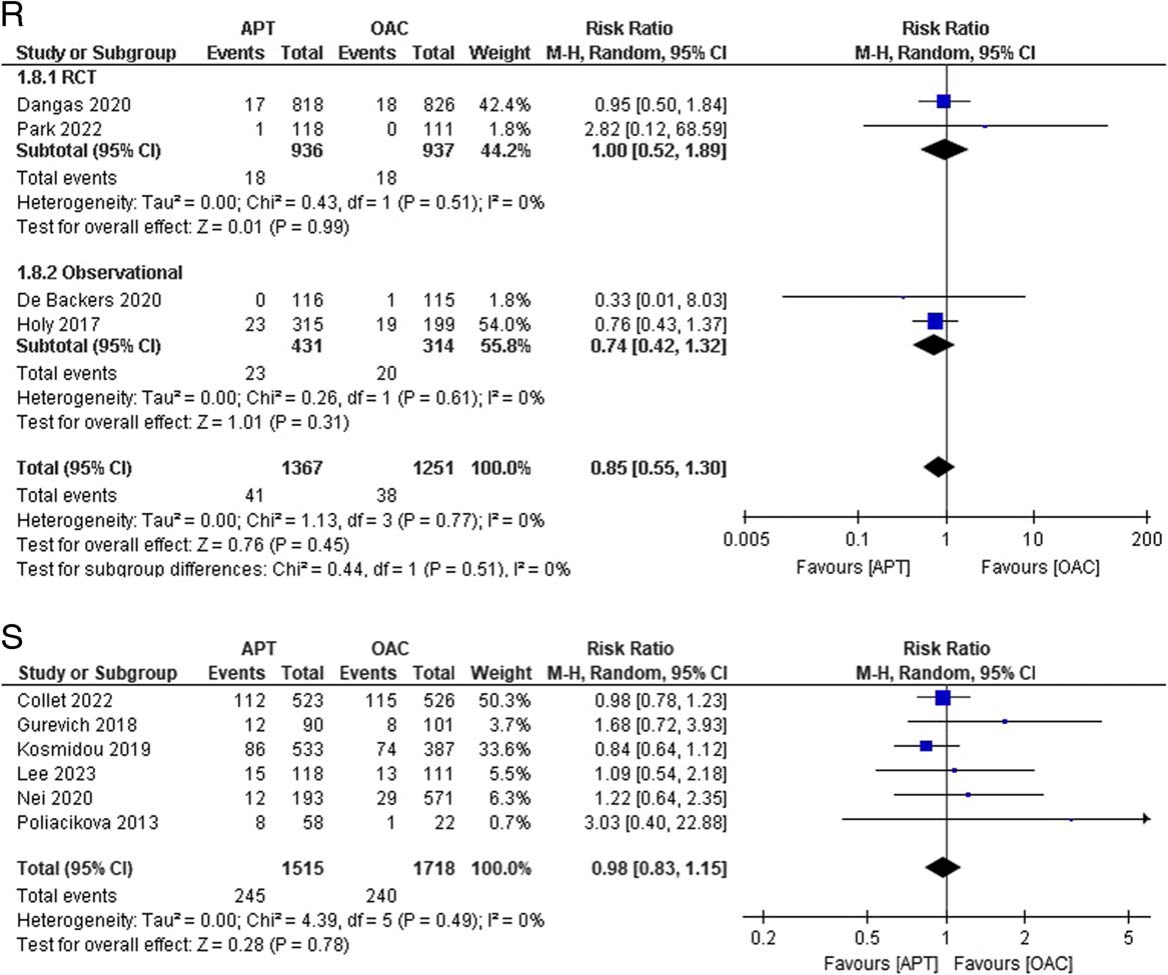

### Secondary outcomes

Cardiovascular-related deaths were reported in five studies^13–15,18,20^. We observed that the risk of cardiovascular-related deaths was lower in the APT group (6.50%) than in the OAC group (6.53%) [RR:0.91; 95% CI:0.73–1.14; *P*=0.42; I^2^ = 0%] (Fig. 2C). No heterogeneity was observed among the studies. Subgroup analysis was conducted, and heterogeneity remained 0% for both subgroups, with the results remaining statistically insignificant (Fig. 2D). Only three studies reported the incidence of MI^15,16,21^, with no difference in risk between the APT and OAC groups [2.32% versus 2.20%; RR:1.69; 95% CI:0.43–6.72; *P*=0.46; I^2^ = 56%] (Fig. 2E). Moderate heterogeneity was observed. We performed leave-one-out sensitivity analysis to address this, and after excluding the study by Dangas *et al*.^14^, no heterogeneity was reported among the studies. Stroke/TIA outcomes were reported in eight studies^14–20,24^. Though the incidence of all-cause stroke was lower in the APT group, we did not observe any statistical difference between the APT and OAC groups in comparison [4.0% versus 4.71%; RR:0.79; 95% CI:0.58–1.06; *P*=0.12; I^2^=0%] (Fig. 2F). Upon performing subgroup analysis based on their study designs, the results remained insignificant with 0% heterogeneity in both the RCT subgroup [RR: 0.89; 95% CI: 0.54–1.46; *P*=0.57, I^2^=0%] and the observational subgroup [RR: 0.74; 95% CI: 0.51–1.07; *P*=0.53; I^2^=0%] (Fig. 2G). The outcome of ischaemic stroke was reported in three studies^14,15,19^. Although the incidence of ischaemic stroke was lower in the APT group than in the OAC group, the difference was not statistically significant [2.63% versus 3.18%; RR:0.83; 95% CI:0.50–1.37; *P*=0.47; I^2^=0%] (Fig. 2H). No heterogeneity was observed among the studies. Haemorrhagic stroke outcomes were reported in two studies^14,19^. The risk was comparable between the two groups [0.33% versus 0.32%; RR:1.08; 95% CI: 0.23–5.15; *P*=0.92; I^2^=0%] (Fig. 2I). No heterogeneity was observed in any of the included studies.

Systemic embolism as an outcome was reported in four studies^14,15,17,21^. No statistically significant difference was observed in the outcome between the APT and OAC groups [0.88% versus 1.79%; RR:0.87; 95% CI:0.44–1.70; *P*=0.68; I^2^=0%] (Fig. 2J). No heterogeneity was observed among the studies. Subgroup analysis was performed, revealing nil heterogeneity, and the results remained insignificant in both the RCT and observational subgroups (Fig. 2K). The outcome of valve thrombosis was reported in 5 studies^13–15,20,23^. The risk of valve thrombosis was higher in the APT group than in the OAC group. We observed statistically significant differences among the groups [4.64% versus 1.65%; RR:2.61; 95% CI:1.56–4.36; *P*=0.0002; I^2^=28%] (Fig. 2L). Moderate heterogeneity was observed among studies. Leave-one-out sensitivity analysis of Collet *et al*.^13^ reduced heterogeneity to 0%. Upon performing subgroup analysis, heterogeneity was observed in the RCT group at 44% (RR: 2.87; 95% CI: 1.40–5.88; *P*=0.004) and 34% in the observational subgroup [RR: 2.69; 95% CI: 0.62–11.60] (Fig. 2M).

### Bleeding events

Five studies reported major bleeding events^14,15,17,18,20^. A lower incidence of bleeding was noted in the APT group than that in the OAC group. However, no statistical significance was observed between the groups [5.21% versus 5.43%; RR:0.79; 95% CI:0.51–1.21; *P*=0.28; I^2^=41%] (Fig. 2N). Moderate heterogeneity was observed among the studies. We performed a leave-one-out sensitivity analysis, decreasing it to 0% when Dangas *et al*.^14^ were excluded. Upon performing subgroup analysis, heterogeneity reduced to 0% in both subgroups, revealing a statistically significant lower risk of major bleeding in the RCT subgroup [RR: 0.50; 95% CI: 0.29–0.87; *P*=0.01; I^2^=0%], but comparable risk in the observational subgroup [RR: 1.04; 95% CI: 0.75–1.44; *P*=0.81; I^2^=0%] (Fig. 2O). Minor bleeding outcomes were reported in three studies^13,15,17^. The risk was comparable between the two groups [10.43% versus 9.57%; RR:1.09; 95% CI: 0.80–1.47; *P*=0.58; I^2^=0%] (Fig. 2P). No heterogeneity was observed among the studies.

The outcome of life-threatening bleeding was reported in four studies^14,15,17,20^. The incidence of life-threatening bleeding was comparable between groups. However, the results were not statistically significant [3.0% versus 3.03%; RR:0.85; 95% CI:0.55–1.30; *P*=0.45; I^2^=0%] (Fig. 2Q). No heterogeneity was noted among the studies. On subgroup analysis, the results remained statistically insignificant with nil heterogeneity across both subgroups (Fig. 2R). Approximately six studies reported the outcome of any bleeding^13,18,19,21,23,24^. The incidence of bleeding was higher in the APT group than in the OAC group, but this finding was also insignificant [16.17% versus 13.97%; RR:0.98; 95% CI:0.83–1.15; *P*=0.78; I^2^=0%] (Fig. 2S).

### Quality assessment and publication bias

**Figure 3:**
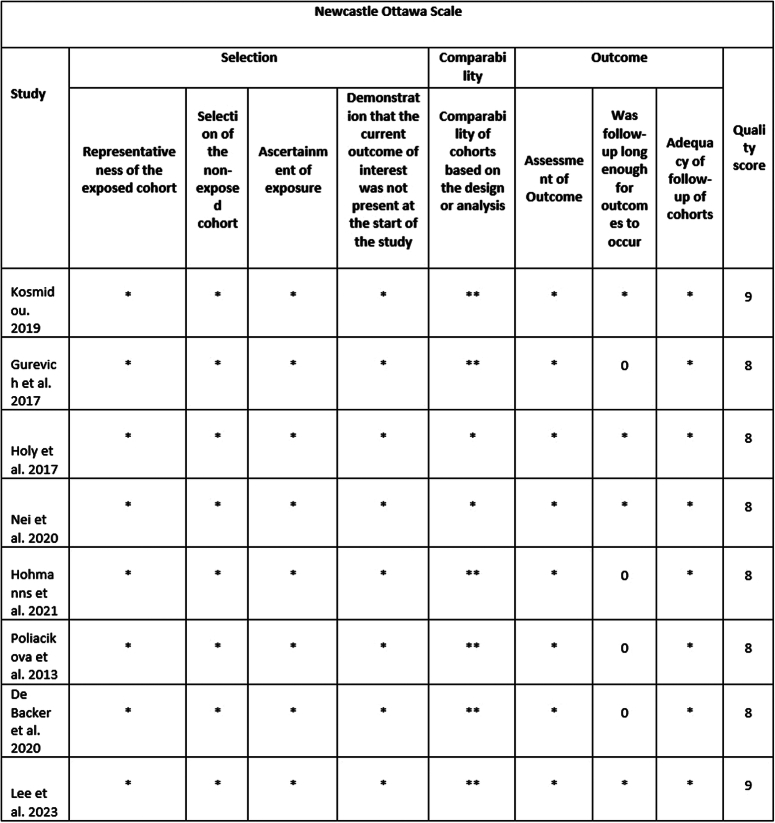
Risk of bias assessment of observational studies using Newcastle–Ottawa Scale (NOS). The following sections in the figures were rated per study and the bias was assessed as: low bias risk (7–10 points), moderate bias risk (4–6 points) and high bias risk (0–3 points).

**Figure 4:**
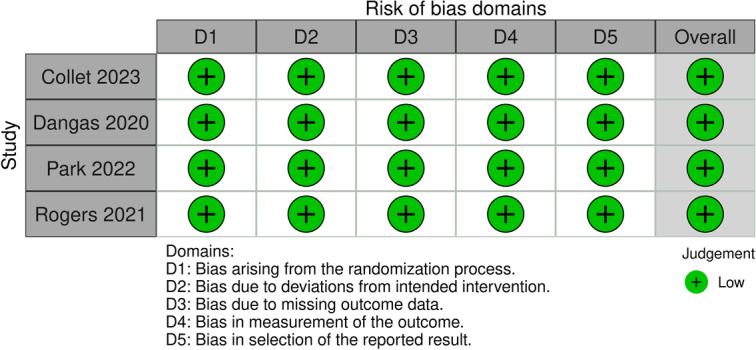
Risk of bias assessment for randomized controlled trials using the Cochrane’s Risk of Bias 2.0 tool.

The Newcastle–Ottawa Scale for observational studies showed a low risk of bias in all the studies (Fig. 3). The findings of the Risk of Bias Assessment Tool 2.0 indicated “low risk” for the RCTs (Fig. 4). A symmetric distribution was observed on visual assessment of the funnel plots, suggesting a low chance of publication bias (Fig. 5).

Figure 5Funnel plots to assess publication bias (A) all-cause mortality; (B) cardiovascular-related death; (C) myocardial infarction; (D) stroke/TIA; (E) ischaemic stroke; (F) haemorrhagic stroke; (G) any bleeding; (H) life-threatening bleeding; (I) major bleeding; (J) minor bleeding; (K) systemic embolism; (L) valve thrombosis. RR, risk ratio.

**Figure:**
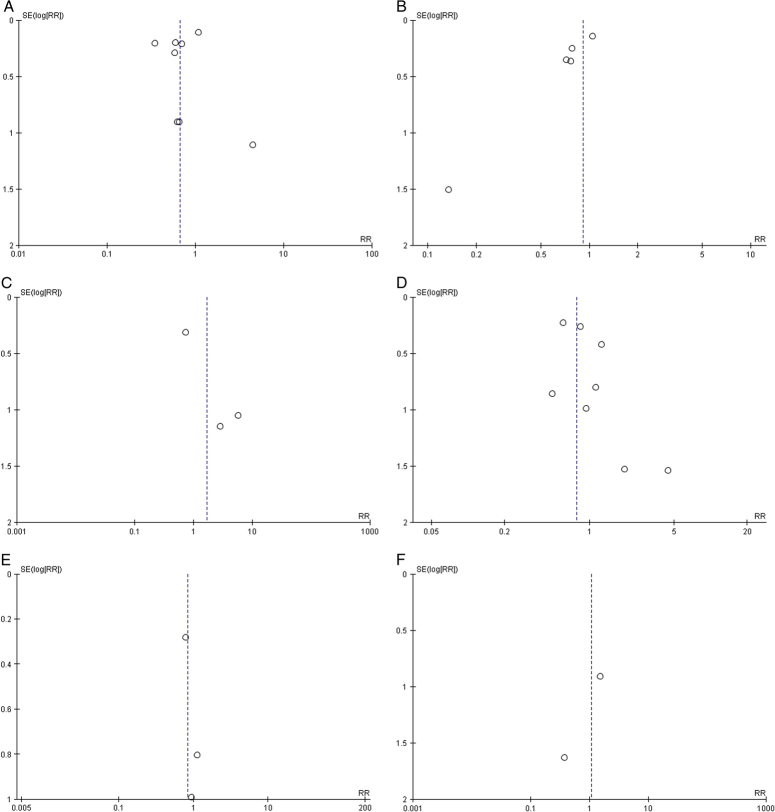

**Figure:**
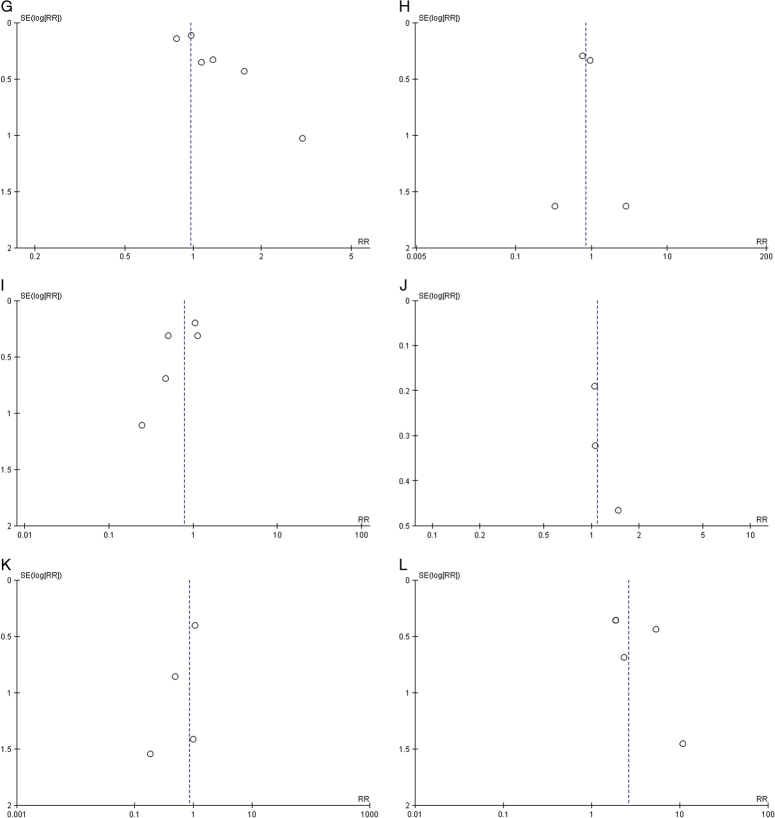

## Discussion

Our analysis revealed no significant differences in ACM, cardiovascular death, MI, stroke/TIA, ischaemic stroke, haemorrhagic stroke, any bleeding, life-threatening bleeding, minor bleeding, or systemic embolism in post-TAVR patients receiving APT and NOACs. Interestingly, we did observe an increased risk of valve thrombosis in the APT group, favoring the use of NOACs over APT.

With technological advancements over the past decade, TAVR has become increasingly common for patients with symptomatic and severe aortic stenosis and high surgical risk. While mortality associated with TAVR is decreasing, concerns regarding ischaemic and haemorrhagic complications and their prevention through optimal antithrombotic therapy persist^25–27^.

DAPT (consisting of clopidogrel and aspirin) is recommended as an empirical treatment for patients without any indication for anticoagulation, according to ACC guidelines^28^. Alternatively, anticoagulation is recommended with VKAs to achieve an INR of 2.5 in low-risk patients for about three months^28^. The ESC recommends DAPT for the first 3–6 months, followed by SAPT for life^29^. However, a detailed guideline for OAC for TAVR patients, especially for those with indications, remains ambiguous^30^. Additionally, clinical trials and observational studies have investigated the superiority of APT or OAC as the most suitable regimen for managing post-TAVR care and complications.

Numerous RCTs have been recently published, analyzing APT treatment and OAC as control groups. Collet and colleagues reported that while bleeding endpoints were similar between Apixaban and APT groups, they observed a concerning excess of non-cardiovascular deaths with apixaban, an association our study did not demonstrate. Conversely, they noted a significant reduction in obstructive valve thrombosis and venous thromboembolic events in patients receiving Apixaban compared to APT, which is in line with the findings of our own study^13^. Dangas and colleagues observed higher rates of death or thromboembolic complications in the Rivaroxaban group and higher rates of bleeding complications. Dangas highlighted the challenge of Rivaroxaban in the elderly population with multiple comorbidities when it came to haemorrhagic and thromboembolic events^14^. This outweighed the benefit of Rivaroxaban, even though it was associated with a lower incidence of subclinical valve-leaflet thickening and reduced leaflet motion compared to APT, as indicated by the imaging sub-study GALILEO^17^. However, Dangas *et al*.^14^ had a limitation: the increased number of deaths in the rivaroxaban group, compared to the APT, did not seem to be directly attributed to bleeding events, with most fatalities occurring long after discontinuation of the trial drug. The ADAPT-TAVR trial compared Edoxaban with DAPT to prevent leaflet thrombosis and associated risks of cerebral thromboembolism and neurological dysfunction. It evaluated data from patients post-TAVR without an indication for oral anticoagulation and found that edoxaban was superior to APT in terms of a lower incidence of leaflet thrombosis^15^. DeBacker *et al*.^17^, a sub-study of a trial, found the superiority of a rivaroxaban-based strategy in preventing subclinical leaflet-motion abnormalities but also indicated a higher risk of death, thromboembolic complications, and bleeding compared to antiplatelet-based strategies. Contrary to the results from these studies, our study did not demonstrate any difference between the two groups when it came to the risk of mortality or bleeding events.

To determine the most appropriate treatment and control group, numerous observational studies also looked at identical treatment and control arms. The effectiveness of OAC alone in preventing stroke following TAVR was called into doubt by Kosmidou *et al*.^18^‘s analysis of the PARTNER 2 cohort, which revealed that while APT, with or without anticoagulant medication, lowered the risk of stroke at two years, OAC alone did not reduce the incidence of stroke at that time. Gurevich and colleagues demonstrated that routine anticoagulation is feasible for most TAVR patients, carrying a similar risk of bleeding and cerebral ischaemia events as that of APT. This calls into question the long-standing practice of DAPT medication and highlights the significance of developing a well-rounded antithrombotic approach to reduce adverse events in the high-risk early post-TAVR phase^19^. Holy and colleagues conducted a retrospective analysis and compared outcomes of two antithrombotic regimens following TAVR. They noted that, at the 1-year follow-up, both DAPT and OAC groups had similar outcomes regarding ACM, myocardial infarction, stroke, clinical valve thrombosis, and life-threatening and major bleeding. However, clinical valve thrombosis was the only incident in the DAPT group, which was then treated with OACs^20^. The findings by Holy and colleagues underscore the findings of our own study. Hohmann *et al*.^22^ reported higher ACM rates in the OAC group. Lee *et al*.^23^ performed a subgroup analysis on the ADAPT-TAVR population and found that the Edoxaban group favored a lower incidence of subclinical leaflet thrombosis and cerebral thromboembolism, and this finding was consistent across multiple clinical and anatomic groups. Poliacikova *et al*.^24^ reported a higher risk of stroke/transient ischaemic attack and bleeding in the APT compared to the warfarin group, but the study had a very limited sample size, which may introduce bias into the results.

Collet and colleagues, Dangas and colleagues, and Holy and colleagues reported complications of bleeding and thromboembolism^13,14,20^, as well as deaths in patients on OACs, whereas they reported that OACs were superior when discussing valvular complications like leaflet thickening and valvular thrombosis. An important point to consider is that Dangas *et al*.^14^ attributed mortality to the frailty of the study population. Hohmann *et al*.^22^ studied ACM in similar treatment and control arms and reported that APT is superior. DeBacker *et al*.^17^ reported a higher incidence of death, thromboembolic complications, and bleeding in the OAC group. Rogers *et al*.^16^ reported lower ACM, stroke, and myocardial infarction in the OAC group, which was receiving both warfarin and aspirin. Nei and colleagues and Guverich and colleagues reported non-superiority of both regimens^19,21^. Lee and colleagues and Park and colleagues also reported an increased incidence of valve thrombosis in the OAC group, consistent with our own findings^15,23^. An important observation was that even though the OAC group had lower risk of valve thrombosis, this did not translate to significantly affecting the risk of other clinical outcomes, thus challenging the clinical relevance of the severity of an increased risk of valvular thrombosis with APT.

### Limitations

Our meta-analysis has certain limitations. First, a significant portion of the included studies were observational and retrospective, which are inherently more prone to contribute to increased heterogeneity. Second, the risk ratios in our study were not adjusted for confounding variables, which may have affected the results. Third, our control arm for each study had different OACs, either in conjunction with APT or alone, which may also have contributed to the heterogeneity among the studies. A subgroup analysis was warranted; however, owing to the limited number of studies, it could not be performed. Finally, our study did not specify indications for OAC use. These indications vary from atrial fibrillation to thromboembolism. Therefore, heterogeneity should be considered when interpreting these results.

## Conclusions

Our study demonstrated an increased risk of valve thrombosis in patients who received APT after TAVR. However, no differences in the risk of ACM, cardiovascular death, MI, stroke/TIA, ischaemic stroke, haemorrhagic stroke, or any bleeding events were observed between the two groups. Therapy after TAVR should thus be based on whether any indications for the use of OAC exist, the risk of valvular thrombosis present in the patient groups, and if any contraindications to either class exist. Studies comparing the efficacy of specific APTs to specific OACs are needed to generate real-world clinically relevant data about the superiority of one drug class over the other. Furthermore, large-scale RCTs are required to generate more robust data.

## Ethical considerations

No ethical approval was required for this study design as all data was taken from publicly available data.

## Consent

No consent was required due to retrospective review nature of study from public information.

## Source of funding

The authors have no funding to report

## Author contribution

A.G.: conceptualization, methodology, investigation, supervision, illustrations. F.Q.A., M.D.T., S.G.K., M.C., J.B., D.S., S.A.S., M.M.: manuscript writing and editing. M.D.: supervision, investigation, manuscript writing and editing. A.H.S.: conceptualization, methodology, investigation, supervision.

## Conflicts of interest disclosure

The author declares no conflicts of interest.

## Research registration unique identifying number (UIN)

PROSPERO- CRD42023470549.

## Guarantor

A.G.

## Data availability statement

No data were analysed from datasets.

## Provenance and peer review

Not commissioned, externally peer-reviewed.

## Supplementary Material
