# Supplementary material for: Efficacy and outcomes of antiplatelet therapy versus oral anticoagulants in patients undergoing transcatheter aortic valve replacement: a systematic review and meta-analysis
Source: Ann Med Surg (Lond). 2024 Mar 15;86(5):2911–25. doi: 10.1097/MS9.0000000000001908 (PMC11060210; doi:10.1097/MS9.0000000000001908)
Supplement: Supplementary file 3 [file ms9-86-2911-s003.docx]

Online supplementary file:

Complete Database search strategy:

(((((Transcatheter Aortic Valve Replacement) OR (Transcatheter aortic valve implantation)) OR (TAVI)) OR (TAVR)) AND (((((((((Anti-Platelet Therapy, Dual) OR (Anti-Platelet Therapies, Dual)) OR (Dual Anti Platelet Therapy)) OR (Dual Anti-Platelet Therapies)) OR (Antiplatelet therapy)) OR (APT)) OR (DAPT)) OR (aspirin)) OR (clopidogrel))) AND ((((((((((((((((((((((((((((((((((((((((((Eliquis) OR (BMS 562247)) OR (BMS562247)) OR (BMS-562247-01)) OR (BMS-562247)) OR (apixaban)) OR (edoxaban)) OR (N-(5-chloropyridin-2-yl)-N'-((1S,2R,4S)-4-(N,N-dimethylcarbamoyl)-2-(5-methyl-4,5,6,7- tetrahydro(1,3)thiazolo(5,4-c)pyridine-2-carboxamido)cyclohexyl)oxamide)) OR (N-(5-chloropyridin-2-yl)-N'-((1S,2R,4S)-4-(N,N-dimethylcarbamoyl)-2-(5-methyl-4,5,6,7-tetrahydrothiazolo(5,4-c)pyridine-2-carboxamido)cyclohexyl)ethanediamide p-toluenesulfonate monohydrate)) OR (Savaysa)) OR (DU-176)) OR (DU-176b)) OR (edoxaban tosylate)) OR (N-((2-(((4-(aminoiminomethyl)phenyl)amino)methyl)-1-methyl-1H-benzimidazol-5-yl)carbonyl)-N-2-pyridinyl-beta-alanine)) OR (Dabigatran Etexilate)) OR (Etexilate, Dabigatran)) OR (Dabigatran Etexilate Mesylate)) OR (Warfarin)) OR (4-Hydroxy-3-(3-oxo-1-phenylbutyl)-2H-1-benzopyran-2-one)) OR (Apo-Warfarin)) OR (Aldocumar)) OR (Gen-Warfarin)) OR (Warfant)) OR (Warfarin Potassium)) OR (Potassium, Warfarin)) OR (Warfarin Sodium)) OR (DCP (prothrombin)) OR (decarboxyprothrombin)) OR (des(gamma-carboxy)prothrombin)) OR (des-gamma-carboxy prothrombin)) OR (non-carboxylated factor II)) OR (PIVKA II)) OR (PIVKA-II (protein induced by vitamin K absence or antagonist-II)) OR (acarboxy prothrombin)) OR (descarboxylated prothrombin)) OR (prothrombin precursor)) OR (isoprothrombin)) OR (Oral Anticoagulant, Direct-Acting)) OR (DOAC)) OR (Direct Acting Oral Anticoagulant)) OR (Factor Xa Inhibitor)) OR (oral anticoagulants))
